# Rituximab Therapy in Refractory Ocular Cicatricial Pemphigoid: A Case Report

**DOI:** 10.3390/reports8030115

**Published:** 2025-07-20

**Authors:** Sania Vidas Pauk, Antonela Geber, Iva Bešlić, Ines Lakoš-Jukić, Tomislav Kuzman

**Affiliations:** 1 Department of Ophthalmology, University Hospital Centre Zagreb, School of Medicine, University of Zagreb, 10000 Zagreb, Croatia; saniavidaspauk@kbc-zagreb.hr (S.V.P.); iva.beslic@kbc-zagreb.hr (I.B.); tomislav.kuzman@kbc-zagreb.hr (T.K.); 2 Department of Dermatovenereology, University Hospital Centre Zagreb, School of Medicine, University of Zagreb, 10000 Zagreb, Croatia; ines.lakos.jukic@kbc-zagreb.hr

**Keywords:** mucous membrane pemphigoid, ocular cicatricial pemphigoid, biologic therapy, rituximab

## Abstract

**Background and Clinical Significance**: Ocular cicatricial pemphigoid (OCP) is a rare autoimmune disease affecting the conjunctiva and oral mucosa. Chronic inflammation causes conjunctival scarring, leading to symblepharon, trichiasis, corneal damage, and possible blindness. Diagnosis is clinical, supported by biopsy and immunofluorescence. Treatment includes systemic corticosteroids, immunosuppressants, and biologics in refractory cases. **Case Presentation**: A 64-year-old male presented with ocular irritation, trichiasis, and counting fingers (CF) visual acuity in the left eye. Slit-lamp examination revealed conjunctival inflammation, corneal epithelial defect, and symblepharon in the left eye. Biopsy confirmed ocular cicatricial pemphigoid (OCP). He was treated with topical steroids, cyclosporine, subconjunctival injections, and systemic corticosteroids, followed by surgery, which improved BCVA to 0.10 logMAR. Two years later, disease progression resulted in severe inflammation and visual decline in both eyes. Systemic azathioprine and corticosteroids achieved partial control. Due to insufficient response, rituximab therapy was initiated, leading to significant reduction in inflammation and stabilization of disease. Right eye BCVA improved to 0.16 logMAR; the left remained at CF. The patient continues to receive rituximab during exacerbations and is under regular follow-up. **Conclusions**: Early diagnosis and timely systemic treatment are essential in preventing vision loss in OCP. In refractory cases, biologic agents like rituximab may offer effective disease control.

## 1. Introduction and Clinical Significance

Mucous membrane pemphigoid (MMP) is a rare systemic condition that mainly affects the conjunctiva and oral mucosa [1,2,3]. Ocular involvement is seen in about 70% of MMP cases and is referred to as ocular cicatricial pemphigoid (OCP). The disease may also affect the nasal mucosa, pharynx, larynx, esophagus, anus, and genital region. It typically presents with mucosal lesions that can lead to progressive scarring. In approximately 15% of cases, skin involvement may occur, particularly on the scalp and face, where scarring can lead to hair loss, known as alopecia. [4,5].

The etiology of OCP remains unclear. It is considered an autoimmune disease characterized by a type II hypersensitivity reaction, in which autoantibodies are directed against epithelial junction proteins such as BP230 (bullous pemphigoid antigen I, a desmoplakin) and BP180 (bullous pemphigoid antigen II, a transmembrane hemidesmosomal protein) [2,4,6,7].

Ocular manifestations may present as chronic conjunctivitis, conjunctival defects, and subepithelial fibrosis, which can progress to conjunctival shrinkage and the development of symblepharon. Potential complications include entropion, trichiasis, corneal erosions, corneal neovascularization, dry eye disease, keratinization of the ocular surface, and secondary glaucoma, all of which can ultimately lead to vision loss or blindness [3,4,5].

The disease usually starts in one eye but tends to progress to bilateral involvement over time. It occurs approximately twice as often in women as in men, with the average age of onset being around 60 years. This chronic condition is marked by alternating phases of remission and exacerbation [4].

The differential diagnosis encompasses a range of systemic and ocular conditions of autoimmune, infectious, inflammatory, or iatrogenic etiology, which can lead to chronic bilateral conjunctivitis and progressive conjunctival fibrosis [7].

Diagnosis is mainly clinical, supported by conjunctival biopsy from affected, scarred tissue and direct immunofluorescence (DFA), which may demonstrate linear deposition along the epithelial basement membrane zone. While a positive biopsy strengthens the diagnosis, a negative result does not definitively rule it out [4,8]. If direct immunofluorescence is negative or cannot be performed, the presence of circulating autoantibodies against epithelial basement membrane components can be assessed in the patient’s serum. This is most commonly carried out using enzyme-linked immunosorbent assay (ELISA) to aid in confirming the diagnosis [9].

The standard therapeutic strategy involves the use of systemic corticosteroids and steroid-sparing immunosuppressants such as azathioprine or cyclophosphamide [2,3,10]. Dapsone may also be beneficial in the management of cicatricial pemphigoid, especially in patients who do not respond adequately to systemic corticosteroids or in whom steroid therapy must be discontinued due to side effects [3]. For refractory cases, additional options include intravenous immunoglobulin (IVIG) and biologic therapies such as anti-TNF agents (etanercept and infliximab), IL-2 antagonists (daclizumab), and the anti-CD20 antibody (rituximab) [11]. Topical treatment includes lubricants and corticosteroids, as well as cyclosporine and tacrolimus in some cases [10,12].

Surgical treatment may involve inferior eyelid retraction plication as a method for correcting entropion and the resulting trichiasis [13,14]. In cases of advanced symblepharon, fornix reconstruction using amniotic membrane transplantation (AMT) may be necessary. Additionally, complications such as persistent corneal ulcers may also require AMT for ocular surface restoration [15]. Additionally, osteo-odonto-keratoprosthesis offers a promising option for preserving limited vision in end-stage disease [2,16]. Management of secondary glaucoma in these patients is challenging due to the presence of ocular surface disease (OSD), which complicates both medical and surgical treatment options [1,17].

## 2. Case Presentation

This case report describes a 64-year-old male patient, currently under regular follow-up at the University Hospital Centre Zagreb, whose condition was retrospectively analyzed. In April 2018, the patient was referred by his primary care physician for dermatologic and ophthalmologic evaluation. He was first seen by a dermatologist, who documented bleeding ulcerations and bullous lesions in the oral cavity, addressed the ocular complaints (burning, photophobia, itching, and tearing), and referred the patient for further ophthalmologic assessment, which was performed shortly thereafter.

His medical history revealed that, in December 2017, he was evaluated by an oral pathology specialist and treated with systemic corticosteroids, which led to improvement of his oral symptoms, including gingival bleeding and erosions that had persisted for five years. He also suffered from frequent nosebleeds, which were managed with cauterization by an otorhinolaryngologist. Ophthalmologically, he had recurrent conjunctivitis and trichiasis for the past year, both treated by a general ophthalmologist. Despite treatment with antibiotics and corticosteroids, the conjunctivitis showed no improvement, while the trichiasis was addressed with multiple sessions of electroepilation.

At the initial slit-lamp examination, the left eye showed conjunctival injection, a central corneal epithelial defect, and conjunctival shortening mainly in the inferior fornix, accompanied by symblepharon formation. The right eye was unaffected at this time. The best corrected visual acuity (BCVA) in the left eye was counting fingers (CF) at 1 m. No active lesions were observed in the oral mucosa at that stage.

A biopsy taken from the inferior conjunctival fornix confirmed the diagnosis of OCP. DFA demonstrated linear deposits of IgG and C3 along the epithelial basement membrane zone. ELISA was negative for desmoglein (DSG) 1 and 3, which were tested as part of the dermatological workup to exclude pemphigus diseases, as well as for BP230, but a positive result was found for BP180.

Initial treatment of the left eye involved topical lubricants, corticosteroid and cyclosporine eye drops, and multiple subconjunctival injections, combined with systemic corticosteroid therapy. Trichiasis was addressed through electroepilation of both eyelids and inferior eyelid retractor plication on the left side. Additionally, AMT was performed to reconstruct the fornix, which led to an improvement in the BCVA of the left eye to 0.6 decimal Snellen (logMAR 0.22). Subsequently, oral dapsone and doxycycline were introduced into the treatment regimen, resulting in a further increase in BCVA to 0.8 decimal Snellen (logMAR 0.10).

**Figure 1:**
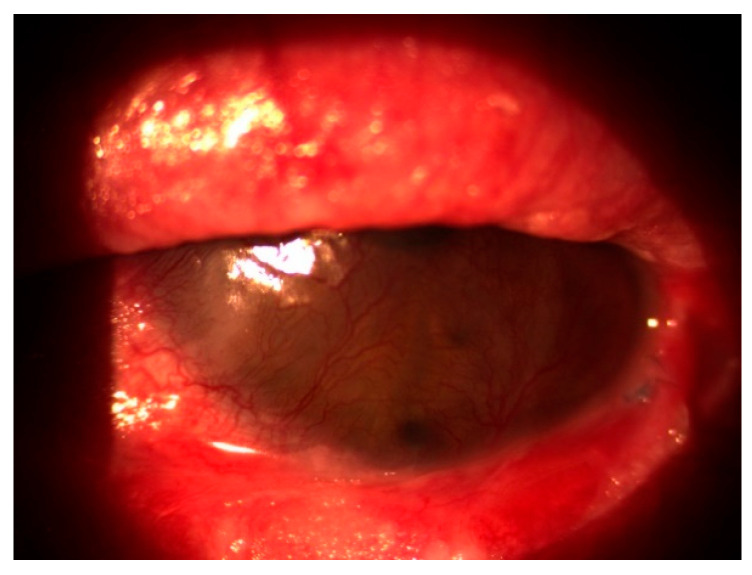
Severe inflammation with corneal neovascularization, keratinization, and ankyloblepharon in the left eye.

**Figure 2:**
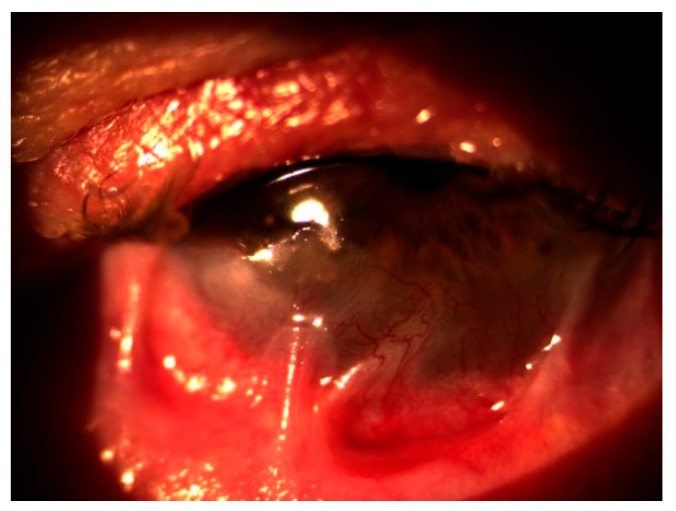
Conjunctivitis with symblepharon in the right eye.

In October 2020, the patient returned with a best corrected visual acuity (BCVA) of hand movements (HM) in the left eye and newly developed involvement of the right eye, which had a BCVA of 0.8 decimal Snellen (logMAR 0.10) at that time. Slit-lamp examination showed severe inflammation accompanied by neovascularization, keratinization of the ocular surface, and ankyloblepharon in the left eye (Figure 1), as well as conjunctivitis with symblepharon formation in the right eye (Figure 2).

Systemic corticosteroid therapy was started, accompanied by immunosuppressive treatment with azathioprine. Topical management included artificial tears, ointments, autologous serum eye drops, and cyclosporine drops. Despite several months of therapy, the BCVA remained at CF at 1 m in the left eye and 0.5 decimal Snellen (logMAR 0.30) in the right eye, with ongoing signs of active inflammation.

To summarize the course of systemic treatment, dapsone and doxycycline were prescribed as maintenance therapy. However, the patient was subsequently lost to follow-up, and it remains unclear for how long this regimen was continued. Upon returning with clinical worsening, systemic corticosteroids combined with azathioprine were introduced and maintained for nearly 9 months. Due to further disease progression despite this therapy, biologic treatment with rituximab was initiated.

**Figure 3:**
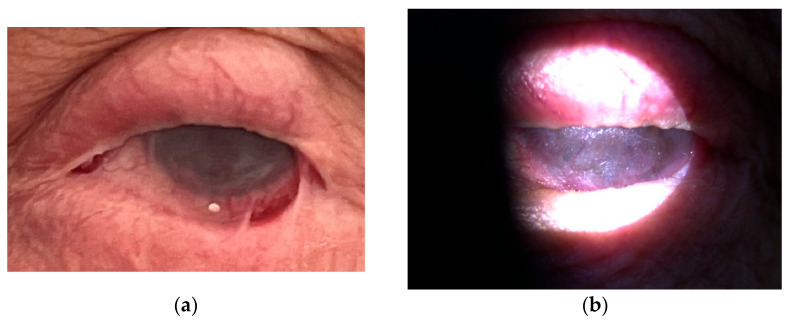
(**a**,**b**) Left eye after rituximab therapy.

**Figure 4:**
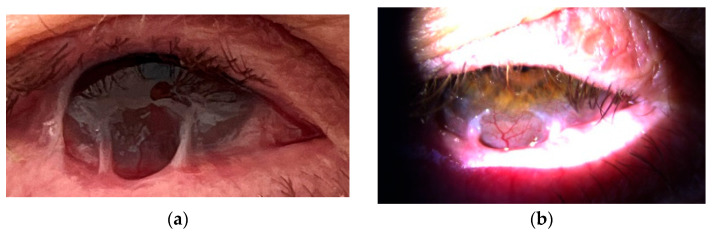
(**a**,**b**) Right eye after rituximab therapy.

Therapy with the anti-CD20 monoclonal antibody rituximab (RTX) was started in July 2021. During hospitalizations, the patient received a cycle of intravenous RTX, consisting of two infusions of 1000 mg administered biweekly on days 0 and 14. Premedication included paracetamol 500 mg orally, solumedrol 125 mg intravenously, and calcium gluconate 10% (10 mL) intravenously. Each rituximab dose was diluted in 1000 mL of 0.9% NaCl and infused over 4–5 h. Following treatment, the patient underwent frequent follow-up evaluations weekly, which included visual acuity testing, clinical grading of conjunctival inflammation, and assessment for symblepharon formation. In the case of clinical exacerbation, the same rituximab cycle (two infusions at a two-week interval) was repeated. After six months, slit-lamp examination showed effective control of active conjunctival inflammation, although the existing cicatricial changes remained stable without further progression (Figure 3 and Figure 4). The patient reported a marked reduction in subjective symptoms following the initiation of biologic therapy. BCVA in the left eye remained at CF at 1 m, while the right eye improved to 0.7 decimal Snellen (logMAR 0.16) after treatment.

Subsequently, the patient received multiple courses of rituximab therapy during disease exacerbations, in combination with corticosteroids and azathioprine. The condition has been stabilized without additional vision loss. However, further surgical procedures will be necessary to address scar tissue changes that arose during the active phase. The patient continues to be followed regularly. The current follow-up schedule includes visits every six months, with instructions to report earlier if symptoms worsen. Maintenance therapy includes topical cyclosporine once daily; autologous serum drops six times daily; intensive lubrication with artificial tears and corneal gel; and systemic therapy with prednisone 10 mg daily, pantoprazole 10 mg daily for gastric protection, and azathioprine 100 mg daily. During follow-up, BCVA has remained stable in both eyes.

At the time of diagnosis and throughout follow-up, the patient remained systemically healthy, with no chronic comorbidities or concurrent medications aside from immunosuppressive therapy prescribed for OCP. Throughout the course of rituximab treatment, the patient did not experience any documented side effects or adverse reactions.

The timeline of therapeutic interventions, alongside corresponding changes in BCVA, is presented in Table 1 to provide a clearer overview of the clinical course and treatment response.

## 3. Discussion

OCP is a complex autoimmune disorder that poses significant challenges in both diagnosis and management. Patients frequently present with nonspecific symptoms, such as ocular irritation or chronic conjunctivitis unresponsive to standard treatments. These early signs are often misinterpreted as milder conditions, potentially leading to delays in accurate diagnosis and appropriate therapy [18].

Diagnosis can sometimes be wrongly dismissed because of negative biopsy results [19,20]. In this case, the diagnosis was confirmed with positive linear staining of the BMZ on DFA analysis. However, it is important to recognize that the sensitivity of DFA can be as low as 50%, particularly in cases with extensive scarring where immunoreactants may be diminished or absent [21,22,23]. Therefore, a diagnosis of OCP should not be excluded prematurely if DFA results are negative, especially when clinical signs and symptoms strongly suggest the disease.

According to the literature, systemic corticosteroids are frequently effective for rapid symptom control but are insufficient for long-term immunosuppression and carry risks of adverse effects with prolonged use [24]. Topical therapies, including lubricants, corticosteroids, and immunomodulatory agents, may promote healing and help reduce ocular surface inflammation [25,26]. Also, intralesional subconjunctival corticosteroid injections have been demonstrated in the literature to effectively reduce the required dosage of systemic corticosteroids [27]. Surgical procedures may be required in some cases; however, they should be delayed until active inflammation is well controlled, since any conjunctival manipulation carries the risk of worsening conjunctival scarring [13,14,15]. In this case, subconjunctival injections were administered alongside systemic and topical corticosteroids as part of the initial treatment to reduce inflammation, allowing for subsequent surgical procedures to be performed.

Dapsone is commonly employed as a first-line therapy in moderate cases, particularly for patients who do not respond to systemic corticosteroids or who must stop corticosteroid treatment due to adverse effects [3].

Numerous immunosuppressive agents, including azathioprine and cyclophosphamide, have been utilized in patients with progressive disease, showing varying degrees of success according to the literature [2,3,10]. In this case, oral azathioprine, when combined with systemic corticosteroids, led to some improvement but provided only limited control over the active conjunctival inflammation.

IVIG therapy has shown some efficacy in refractory cases, although its use is often limited by high cost [28,29]. Among biologic agents, rituximab has emerged as a promising option in patients unresponsive to conventional immunosuppression. In this case, rituximab was initiated after failure of standard treatment, leading to a marked reduction in subjective symptoms and stabilization of ocular signs. Several studies have also evaluated its use in earlier disease stages, suggesting that prompt control of inflammation may help preserve vision and reduce the need for additional immunosuppressive therapy [11,29,30,31].

Several important limitations must be considered when interpreting the findings presented in this case report. As a single-patient observation without a control group or comparator cases, it cannot provide generalizable conclusions about treatment efficacy or long-term outcomes. While an association between rituximab administration and disease stabilization was observed, causality cannot be definitively established. Additionally, the potential for selection bias must be considered, as patients with more favorable outcomes are more likely to be reported in the literature.

However, this case report highlights the importance of a multidisciplinary approach. Close collaboration among ophthalmologists, dermatologists, oral pathologists, and otorhinolaryngologists is essential due to the complex nature of OCP. Given the reported recurrence rate of approximately 22%, regular follow-up is vital to detect disease relapse and manage potential complications [32]. Recent studies suggest that patients treated with biologic agents may have lower recurrence rates [33]. However, careful monitoring is still necessary to promptly identify signs of relapse or complications.

## 4. Conclusions

Early diagnosis and timely systemic treatment of OCP are essential to prevent vision-threatening complications associated with ocular cicatricial pemphigoid. In cases resistant to conventional therapy, biologic agents like rituximab may offer a viable alternative. Furthermore, regular follow-up of this chronic disease can give clinicians the opportunity to stop the irreversible disease progression.

## Figures and Tables

**Table 1:** Timeline of treatment course and corresponding BCVA.

Timepoint	Intervention	BCVA Right Eye	BCVA Left Eye
April 2018	First examination	Unaffected	CF
May–June 2018	LEFT EYE: Topical therapy, electroepilation, inferior eyelid retractor plication and fornix reconstruction with ATM	Unaffected	logMAR 0.22
July 2018	Prescribed maintenance therapy with oral dapsone and doxycycline	Unaffected	logMAR 0.10
October 2020	Exacerbation after loss to follow-up, starting therapy with corticosteroids with azathioprine	logMAR 0.10	HM
July 2021	After therapy with corticosteroids and azathioprine and before RTX therapy	logMAR 0.30	CF
January 2022	6 months after first cycle of RTX therapy	logMAR 0.16	CF
March 2025	Regular follow-up	logMAR 0.16	CF

## Data Availability

The data supporting the findings of this study are not publicly available due to the presence of personal information and ethical considerations.

## Abbreviations

The following abbreviations are used in this manuscript:

AMT	Amniotic Membrane Transplantation
BCVA	Best Corrected Visual Acuity
BMZ	Basement Membrane Zone
CF	Counting Fingers
DFA	Direct Immunofluorescence Analysis
DSG	Desmoglein
ELISA	Enzyme-Linked Immunosorbent Assay
HM	Hand Movements
IL-2	Interleukin-2
IVIG	Intravenous Immunoglobulin
MMP	Mucous Membrane Pemphigoid
OCP	Ocular Cicatricial Pemphigoid
OSD	Ocular Surface Disease
RTX	Rituximab
TNF	Tumor Necrosis Factor
